# Human chorionic gonadotropin (hCG) concentrations during the late first trimester are associated with fetal growth in a fetal sex-specific manner

**DOI:** 10.1007/s10654-016-0201-3

**Published:** 2016-10-05

**Authors:** Mirjana Barjaktarovic, Tim I. M. Korevaar, Vincent W. V. Jaddoe, Yolanda B. de Rijke, Theo J. Visser, Robin P. Peeters, Eric A. P. Steegers

**Affiliations:** 1 The Generation R Study Group, Departments of Internal Medicine, Erasmus Medical Center-Sophia Children’s Hospital, Rotterdam, The Netherlands; 2 Departments of Internal Medicine, Erasmus Medical Center-Sophia Children’s Hospital, Rotterdam, The Netherlands; 3 Rotterdam Thyroid Center, Erasmus Medical Center-Sophia Children’s Hospital, Rotterdam, The Netherlands; 4 Epidemiology, Erasmus Medical Center-Sophia Children’s Hospital, Rotterdam, The Netherlands; 5 Departments of Clinical Chemistry, Erasmus Medical Center-Sophia Children’s Hospital, Rotterdam, The Netherlands; 6 Obstetrics and Gynecology, Erasmus Medical Center-Sophia Children’s Hospital, Wytemaweg 80, 3015 CN Rotterdam, The Netherlands; 7 Pediatrics, Erasmus Medical Center-Sophia Children’s Hospital, Rotterdam, The Netherlands

**Keywords:** hCG, Fetal growth, Birth weight, Fetal sex, Trophoblast

## Abstract

Human chorionic gonadotropin (hCG) is a pregnancy-specific hormone that regulates placental development. hCG concentrations vary widely throughout gestation and differ based on fetal sex. Abnormal hCG concentrations are associated with adverse pregnancy outcomes including fetal growth restriction. We studied the association of hCG concentrations with fetal growth and birth weight. In addition, we investigated effect modification by gestational age of hCG measurement and fetal sex. Total serum hCG (median 14.4 weeks, 95 % range 10.1–26.2), estimated fetal weight (measured by ultrasound during 18–25th weeks and >25th weeks) and birth weight were measured in 7987 mother–child pairs from the Generation R cohort and used to establish fetal growth. Small for gestational age (SGA) was defined as a standardized birth weight lower than the 10th percentile of the study population. There was a non-linear association of hCG with birth weight (*P* = 0.009). However, only low hCG concentrations measured during the late first trimester (11th and 12th week) were associated with birth weight and SGA. Low hCG concentrations measured in the late first trimester were also associated with decreased fetal growth (*P* = 0.0002). This was the case for both male and female fetuses. In contrast, high hCG concentrations during the late first trimester were associated with increased fetal growth amongst female, but not male fetuses. Low hCG in the late first trimester is associated with lower birth weight due to a decrease in fetal growth. Fetal sex differences exist in the association of hCG concentrations with fetal growth.

**Electronic supplementary material:**

The online version of this article (doi:10.1007/s10654-016-0201-3) contains supplementary material, which is available to authorized users.

## Introduction

Optimal intrauterine conditions are essential for proper fetal development and growth. Intrauterine conditions are highly dependent on the placental function since the placenta is the main source of fetal nourishment and the main regulator of the intrauterine environment [1, 2]. A suboptimal intrauterine environment leads to fetal adaptations that may affect fetal growth and thereby lead to lower birth weight [3, 4]. Low birth weight is an important determinant of child’s health and a major risk factor for several noncommunicable diseases later in life including coronary heart disease, stroke, hypertension and type 2 diabetes [5, 6].

Human chorionic gonadotropin (hCG) is a pregnancy-specific hormone that is produced by trophoblast cells from the time of embryo implantation onwards [7]. Besides promoting progesterone production by corpus luteal cells, hCG has been shown to regulate many processes that are related to fetal growth including trophoblast differentiation, uterine growth, various aspects of placentation as well as uterine angiogenesis and vasculogenesis [7, 8]. More specifically, hCG has also been shown to stimulate the production of endocrine gland-derived vascular endothelial growth factor (EG-VEGF), which is involved in the physiology of placental development [9, 10]. By acting on cytotrophoblast cells, EG-VEGF is involved in the process of trophoblast shell and arterial plugs formation, necessary for preventing maternal blood flow into the intervillous space during early pregnancy [9]. hCG may also directly influence uterine and fetal growth by acting on gonadotropin receptors present in the uterine tissue and fetal membranes [8, 11, 12].

Although clinical studies have shown that hCG is associated with adverse outcomes, this association seems to differ according to the gestational age at which hCG is measured. Low hCG concentrations in the first trimester as well as high hCG concentrations in the second trimester have both been associated with pregnancy loss and preeclampsia [13–16]. High hCG concentrations in the second trimester have also been associated with gestational hypertension, fetal growth restriction, fetal death and preterm delivery [16, 17]. Given that hCG concentrations vary throughout gestation, it is remarkable that very little is known about potential gestational time-dependent effects of hCG on adverse pregnancy outcomes.

Given the important role of hCG in many fetal growth-related processes, we examined the overall and gestational age-dependent associations of hCG with fetal growth, as well as possible fetal sex-specific differences, in a large population-based prospective cohort study.

## Materials and methods

### Study population

This study was embedded in the Generation R cohort, a population-based prospective study from early fetal life onwards in Rotterdam, the Netherlands [18]. The study was designed to identify early environmental and genetic causes leading to normal and abnormal growth, development and health during fetal life and childhood [18]. In total, 8879 mothers with expected delivery date between April 2002 and January 2006 were enrolled during pregnancy. Total hCG was determined in all first available serum samples and this data, together with data on birth weight, was available in 7987 mother–child pairs. Women with twin pregnancies (N = 90) or in vitro fertilization treatment (N = 38) were excluded from the analysis.

### hCG measurements

Total hCG was measured in serum using a solid-phase two-site chemiluminiscent immunometric assay, calibrated against WHO 3rd IS 75/537, on an Immulite 2000 XPi system (Siemens Healthcare Diagnostics, Deerfield, IL, USA). The assay detects serum intact hCG, hyperglycosated hCG, serum nicked hCG, serum nicked hyperglycosated hCG, serum asialo hCG, serum hCG free β-subunit and serum nicked hCG β. hCG concentrations were transformed to standard deviation scores adjusted to gestational age at blood sampling [19].

### Fetal growth and birth weight

Early fetal growth was estimated by ultrasound measurement of crown-rump length (CRL) in a subset of women (N = 1526) which had a reliable and regular menstrual cycle [18, 19]. CRL values were transformed to standard deviation scores adjusted to gestational age of pregnancy determined according to the last menstrual period (LMP). CRL was measured by ultrasound in early pregnancy, in a true mid-sagittal plane with the genital tubercle and the fetal spine longitudinally in view [20]. The maximum length from cranium to the caudal rump was measured as a straight line [20].

Fetal weight was estimated by ultrasound measurements in the period of 18–25th week of pregnancy (median = 20.5 week, 95 % range 18.5–23.4 week; N = 7471) and after the 25th week of pregnancy (median = 30.3 week, 95 % range 28.3–33.0 week; N = 7641) and estimations for fetal weight were transformed to standard deviation scores adjusted to gestational age of pregnancy determined by crown-rump length and biparietal diameter, as has been described previously [20]. Information on birth weight was obtained from community midwives, obstetricians and hospital registries. Birth weight standard deviations scores, adjusted for gestational age, were constructed using the Niklasson percentile growth curves [21]. Small for gestational age at birth (SGA) was defined as a standardized birth weight lower than the 10th percentile of the study population.

### Covariates

Information on maternal age, smoking status, educational level and ethnicity was obtained by questionnaires during pregnancy. Ethnicity was determined by the country of origin and was defined according to the classification of Statistics Netherlands [18]. Maternal smoking was classified as no smoking, smoking until known pregnancy and continued smoking during pregnancy. Information on fertility treatment, parity, placental weight at birth and sex of the child was obtained from community midwives, obstetricians, and hospital registries. Gestational weight gain was defined as the difference between self-reported maternal weight before pregnancy and maternal weight measured in the third trimester (a sensitivity analysis using maternal weight measured in early pregnancy and in the third trimester did not reveal more confounding potential or explained variability of the model). Free thyroxine (FT4) and thyroid stimulating hormone (TSH) were available in a subset of 5498 pregnant women during early pregnancy [22].

### Statistical analysis

We investigated the association of hCG concentrations with CRL within a subset group of women (with regular cycles and known last menstrual period) and birth weight within the whole group by using multiple linear regression analysis with restricted cubic splines utilizing three knots. Multivariable associations were graphically depicted by plots (main manuscript) and the key β estimates with 95 % confidence intervals are shown in Supplemental Table 3. We tested for effect modification with gestational age at blood measurement and fetal sex by introducing a product interaction term of hCG and gestational age at blood sampling or fetal sex to the model. Given the fetal growth differences across gestational age and between male and female fetuses, a *P* value of <0.15 was considered for stratification. We subsequently stratified the analyses by quintiles of gestational age at blood sampling and in the case of a difference between these time points, further stratification was performed per one or more gestational weeks. To study the association of hCG with the risk of SGA we used multiple logistic regression models with restricted cubic splines utilizing three knots. The association of hCG concentrations with fetal growth throughout gestation was analyzed using unbalanced repeated measurement regression models for which the outcome consisted of standardized estimated fetal weight in the second and third trimester and standardized birth weight. These models take the correlation between repeated measurements of the same subject into account and allow for incomplete outcome data [23]. We used an unstructured covariance matrix with fixed effects, and added the interaction term of hCG with the time component (gestational age) in the models, and adjusted for covariates. Based on the size of effect estimate differences and biological plausibility extracted from the literature, we stratified these analyses in a similar manner as previously described analyses on birth weight. Lower statistical power in subsequent subset analyses and the need for testing three-way interactions were deemed statistically not viable. The hCG cut-off in the repeated measurement analyses was chosen based on the optimal power necessary for the biologically plausible reference group.

All model covariates were selected based on biological plausibility, change in effect estimate of interest or residual variability of the model. All analyses were adjusted for maternal age, smoking status, BMI, parity, educational level, ethnicity, fetal sex, placental weight at birth, gestational age at blood sampling and gestational weight gain of the mother. Placental weight, as a marker for trophoblast cell mass, may be an important determinant of both hCG concentrations [19] and fetal growth and from that reason was adjusted for in the models. Maternal weight gain was taken as a proxy for the potential confounding effects of hyperemesis gravidarum (HG). Gestational weight change is an important clinical marker of the HG effects [24] and the same goes for other symptoms of HG such as reflux/belching, nausea and vomiting—but addition of these factors did not change our models.

For covariates with missing data, multiple imputation according to the Markov Chain Monte Carlo method was used [25]. Five imputed data sets were created and pooled for the analysis. Maternal smoking, education, ethnicity, BMI, parity, placental weight, gestational weight gain and fetal sex were added to the model. Furthermore, we added gestational age at blood sampling, hCG level and maternal FT4 level as prediction variables only. No statistically significant differences in descriptive statistics were found between the original and imputed datasets.

Statistical analyses were performed using Statistical Package of Social Sciences version 21.0 for Windows (SPSS Inc. Armonk, NY), R statistical software with RMS package version 3.2.0 and SAS software for Windows version 9.3.

## Results

**Fig. 1:**
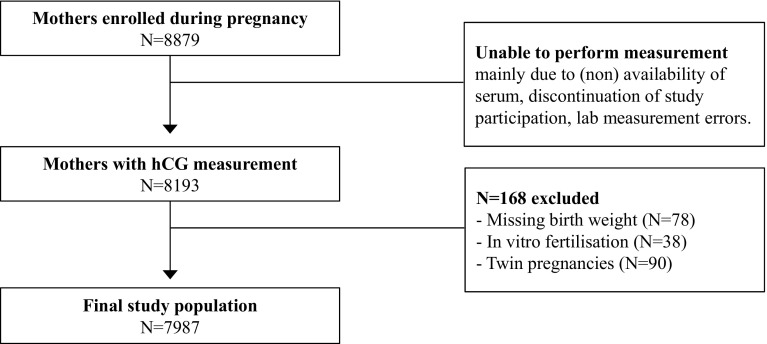
Flowchart showing selection procedure of study population

The final study population consisted of 7987 pregnant women (Fig. 1), descriptive statistics of which are shown in Table 1. Maternal hCG concentrations were measured at the moment of inclusion in the study (median 14.4 weeks, 95 % range 10.1–26.2 weeks). In the study population, the mean (±SD) birth weight was 3412.0 (±559.7) grams, the mean gestational age at birth was 39.8 (±1.9) weeks, the mean maternal age was 29.6 (±5.3) years, women were predominantly nulliparous (55.3 %), non-smokers (72.7 %) and of Dutch origin (46.6 %).

**Table 1:** Descriptive statistics of mother and child pairs

Characteristics	Value
hCG, median (95 % range), IU/l,	35559.0 (6106.8–101808.5)
Birth weight, mean (SD), g	3412.0 (559.7)
Gestational age at blood sampling, median (95 % range), weeks	14.4 (10.1–26.2)
Gestational age at birth, mean (SD), weeks	39.8 (1.9)
Age, mean (SD), years	29.6 (5.3)
BMI, median (95 % range), kg/m^2^	23.9 (18.7–36.5)
*Parity, n (%)*	
Nullipara	4531 (55.3)
Primipara	2475 (30.2)
Multipara	1187 (14.5)
*Smoking status, n (%)*	
Non smokers	5953 (72.7)
Stopped smokers	691 (8.4)
Smokers	1549 (18.9)
*Educational level, n (%)*	
No education or primary education	1046 (12.8)
Secondary education	3844 (46.9)
Higher education	3303 (40.3)
*Ethnicity, n (%)*	
Dutch	3742 (46.6)
Moroccan	530 (6.6)
Turkish	710 (8.8)
Surinam	686 (8.5)
Other-European	598 (7.5)
Other-Non-European	1758 (21.9)
*Fetal sex, n (%)*	
Male	4124 (50.3)
Female	4069 (49.7)
Gestational weight gain, mean (sd), kg	10.0 (5.1)
Placental weight at birth (g), median, (95 % range)	620.0 (390.0–950.0)

### The association of maternal hCG with birth weight

**Fig. 2:**
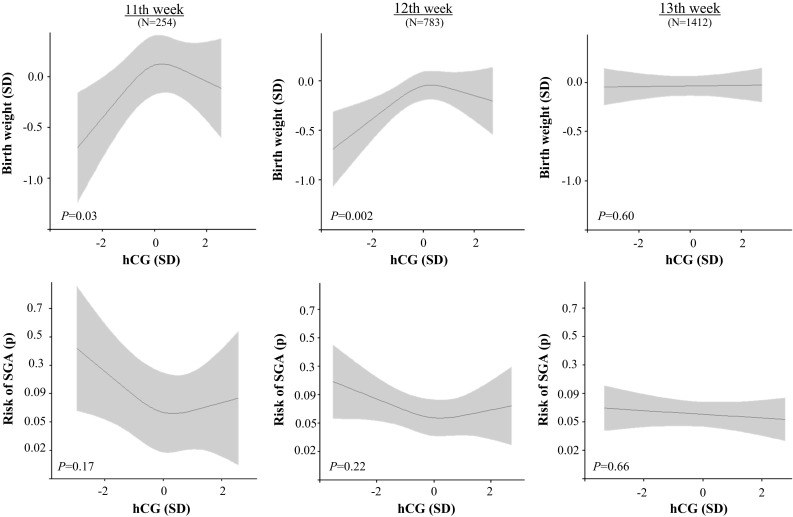
Plots show the linear regression models for total hCG (standardized according to gestational age at measurement; SD) and birth weight (standardized according to gestational age at birth; SD), as well as the logistic regression model for hCG and birth weight small for gestational age (defined as birth weight below 10th percentile for gestational age) as predicted mean with 95 percent confidence interval. Analyses were performed after exclusion of women with IVF treatment (N = 38), twin pregnancy (N = 90) and were adjusted for maternal age, smoking, BMI, parity, placental weight at birth, education level, ethnicity, gestational weight gain and fetal sex

In the whole population, there was a non-linear association of maternal hCG concentrations with birth weight (Supplemental Fig. 1, *P* = 0.009) and the risk of SGA (Supplemental Fig. 1, Supplemental Table 1). Considering that hCG concentrations vary throughout gestation, we investigated whether gestational age at blood sampling modifies the association of hCG concentrations with birth weight. After addition of a product interaction term to the model (hCG*gestational age at blood sampling; *P* = 0.10), we stratified the association of hCG concentration with birth weight by gestational age at blood sampling (Fig. 2; Supplemental Fig. 2). The association of hCG with birth weight was present in the 11th week (Fig. 2, *P* = 0.03; beta estimates shown in Supplemental Table 3) and 12th week of pregnancy (Fig. 2, *P* = 0.002; Supplemental Fig. 3), but not from the 13th week onwards (Fig. 2; Supplemental Fig. 3). The association of hCG with the risk of SGA showed a consistent trend with the results from linear regression analysis with birth weight, with a tendency of higher odds of SGA in low hCG concentrations in the 11th and 12th week (Fig. 2 and Supplemental Fig. 3). The odds of SGA in women with low hCG concentration measured in the 11th and 12th week (<5th to <15th percentile) were 1.80 to 2.21-fold higher than the reference group (Supplemental Table 2).

**Fig. 3:**
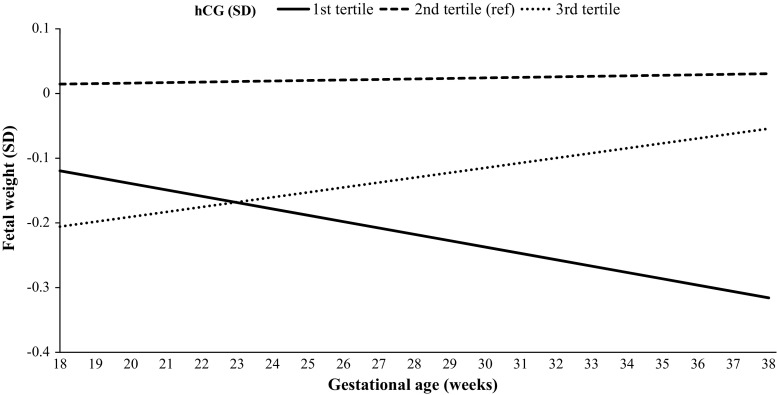
Graph depicts the beta estimates from a repeated measurement model of the association of total hCG (standardized according to gestational age at measurement; SD) with fetal growth (standardized estimated fetal weight measured using ultrasound in mid pregnancy (18–25 weeks), late pregnancy (> 25 weeks) and birth weight). Analyses were performed after exclusion of women with IVF treatment (N = 38), twin pregnancy (N = 90) and were adjusted for maternal age, smoking, BMI, parity, education level, ethnicity, gestational weight gain, placental weight at birth and fetal sex

Subsequently, we set out to investigate if the association of hCG with birth weight occurs due to changes during early pregnancy (crown rump length) or also due to changes in the second half of pregnancy (fetal growth).

### The association of maternal hCG with crown rump length

In the subset of women with regular cycles and known last menstrual period, in which CRL measurement was available, hCG concentrations during early pregnancy were negatively associated with CRL, but this analysis did not reach statistical significance (Supplemental Fig. 4; N = 1526; *P* for interaction fetal sex = 0.10).

### The association of maternal hCG with fetal growth

Maternal hCG concentrations were associated with estimated fetal weight in the whole population, but this association was most prominent in pregnancies in which hCG was measured in the 12th week of gestation (Table 2). The association between hCG concentrations measured in the 12th week of pregnancy and estimated fetal weight is illustrated in Fig. 3. The estimated fetal weight in women with a relatively high hCG concentration (Fig. 3, 3rd tertile, depicted by the dotted line) was lower in mid pregnancy but accelerated throughout gestation, finally reaching a birth weight similar to the reference group (2nd tertile). In contrast, estimated fetal weight in women with a low hCG concentration (Fig. 3, 1st tertile, depicted by the solid line) was similar in mid-pregnancy, but was associated with lower birth weight than the reference group after a decrease of fetal growth in the second half of pregnancy.

**Table 2:** The association of maternal hCG with fetal growth throughout gestation

	Beta	±SE	*P* value	95 % Confidence interval	Number of participants per week
hCG in all	0.002	±0.001	0.0017	(0.001, 0.003)	
hCG stratified per week of measurement					
11th week	−0.001	±0.003	0.81	(−0.007, 0.005)	254
12th week	0.007	±0.002	0.0002	(0.003, 0.010)	783
13th week	0.000	±0.001	0.94	(−0.003, 0.003)	1412
14th week	0.000	±0.002	0.78	(−0.003, 0.004)	1060

Table shows the effects estimates of repeated measurement model for the association of total hCG (standardized according to gestational age at measurement; SD) with fetal growth (standardized estimated fetal weight measured using ultrasound in mid pregnancy (18–25 weeks), late pregnancy (>25 weeks) and birth weight), as beta estimate with standard error. Analyses were performed after exclusion of women with IVF treatment (N = 38), twin pregnancy (N = 90) and were adjusted for maternal age, smoking, BMI, parity, education level, ethnicity, gestational weight gain, placental weight at birth and fetal sex

### Sex-specific differences in the association of hCG with birth weight and fetal growth

Considering the biological differences in fetal growth and in hCG physiology between male and female fetuses, we tested for effect modification by fetal sex. After addition of a product interaction term to the model (hCG*fetal sex; *P* = 0.10), we stratified analyses according to fetal sex. The association of hCG concentrations (measured in week 11–12) and birth weight did not differ for fetal sex (Supplemental Fig. 5 and 6). However, in women with low hCG concentrations (<10th or <15th percentile), the risk of SGA was higher in male than in female fetal-sex (Supplemental Table 4).

**Fig. 4:**
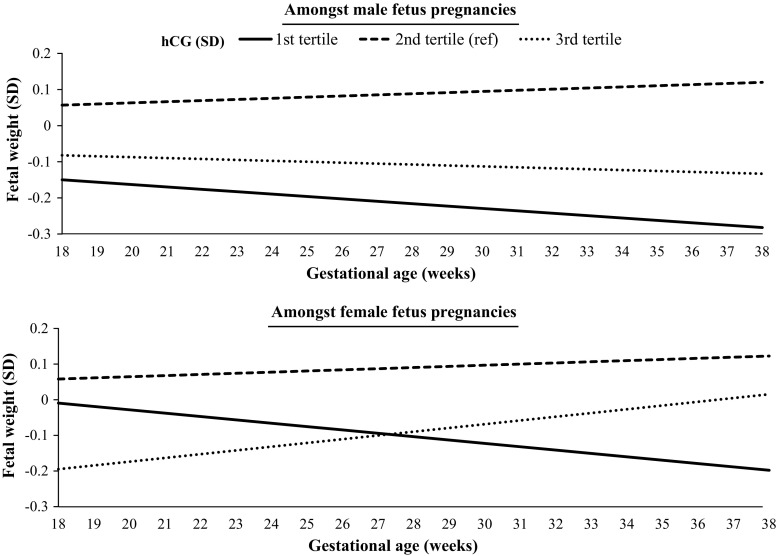
The association of maternal hCG in 11th or 12th week with fetal growth stratified by fetal sex

The association of hCG concentrations with estimated fetal weight differed according to fetal sex (Supplemental Table 5). In women with relatively low hCG during the late first trimester (Fig. 4, 1st tertile, depicted by the solid lines), estimated fetal weight in mid-pregnancy was overall lower in male than in female fetuses. However, in female fetuses, low maternal hCG was associated with a greater deceleration of fetal growth than in male fetuses (−0.17 SD decrease in male fetus pregnancies versus −0.26 SD decrease in female fetus pregnancies; Fig. 4).

In women with high hCG concentrations during the late first trimester (Fig. 4, 3rd tertile, depicted by the dotted lines), estimated fetal weight during mid-pregnancy was overall lower in female than in male fetuses. However, in female fetuses, high maternal hCG concentrations were associated with an acceleration of fetal growth as compared to a slight deceleration in male fetus pregnancies (−0.07 SD decrease in males versus +0.26 SD increase in females; Fig. 4).

There was no effect modification by, or change in the results after adjustment for maternal FT4 and TSH concentrations (data not shown).

## Discussion

Our study shows that low hCG concentrations in the late first trimester are associated with lower birth weight and an increased risk of SGA. We demonstrate that the association of low hCG concentrations in the late first trimester with lower birth weight arises due to a decrease in fetal growth during the second half of pregnancy. In contrast, high concentrations of hCG in the late first trimester were associated with growth acceleration which resulted in a normal birth weight. A low hCG concentration was associated with a decrease in fetal growth, independent of fetal sex. However, high hCG concentrations were associated with an accelerated fetal growth in female, but not in male fetuses.

Fetal growth and birth weight reflect intrauterine conditions during pregnancy. Major healthcare problems such as cardiovascular diseases and type 2 diabetes are preordained in utero and low birth weight is one of the main determinants of those disorders [5, 6]. Previous studies that investigated hCG as a potential determinant of fetal growth restriction mostly focused on either the first or second trimester. Furthermore, most of those studies examined only the association of the β-hCG isoform and fetal growth, meaning that non-measured differences in other important active hCG-isoforms could have influenced the results [26]. A large Danish study of 9450 women in the 8th–13th week of pregnancy reported that a low free β-hCG concentration is associated with SGA [27]. A study of 8012 pregnant women by Krantz et al. reported that a low free β-hCG concentration in the 10th–13th week of pregnancy is associated with an increased risk of fetal growth restriction (FGR) [28]. Amongst 100 women, Abdel Moety et al. also reported that low concentrations of free β-hCG measured in the 11th to 14th week of gestation are associated with FGR [29]. In contrast, other studies showed that a high hCG concentration in the second trimester is associated with a decrease in fetal growth and/or FGR [16, 17]. To date, no study had investigated the association of total hCG with fetal growth or investigated the role of gestational age of hCG measurement and/or fetal sex in this association.

In the current study, low hCG concentrations were only associated with low birth weight when hCG was measured in the 11th or 12th week of gestation, suggesting that hCG has a specific role in fetal growth during the transitional period from the first to the second trimester. This period marks the start of maternal blood supply in the intervillous space and the end of the hypoxic fetal environment [30]. Oxidative stress in early gestation is a risk factor for adverse pregnancy outcomes including fetal growth restriction, which might be due to a lack of antioxidant enzymes in fetal tissues at that time [31]. hCG is indirectly involved in the maintenance of early pregnancy hypoxia via regulating endocrine gland-derived vascular endothelial growth factor (EG-VEGF), a factor that at least partially ensures physiologically low oxygen concentrations during early pregnancy through stimulation of the arterial plugs formation [9]. The specific association in the current study of low hCG in the late first trimester with decreased fetal growth might be due to a suboptimal development of the trophoblast shell and arterial plugs, or an earlier release of the arterial plugs via lower levels of EG-VEGF. This could potentially expose the fetus to the harmful effects of O_2_ free radicals. Further studies are needed to replicate our findings and investigate the association of repeatedly measured hCG concentrations with fetal growth.

Studies have shown that fetal growth and maternal hCG concentrations differ depending on fetal sex. hCG concentrations in pregnancies with a female fetal sex are higher from as early as the third post-fertilization week [32–35]. In this study, the continuous association of hCG concentrations measured at the end of the first trimester with birth weight did not differ between pregnancies with a male or female fetus. Nevertheless, low hCG concentrations were associated with a higher risk of SGA in male fetal sex. This discrepancy already suggests that there is a fetal sex-specific association of hCG with fetal growth patterns. Moreover, further analyses revealed that the association of low hCG during the late first trimester with fetal growth deceleration was stronger in female fetal sex. Furthermore, high hCG concentrations during the late first trimester were associated with growth acceleration in the female, but not male fetuses.

Male fetuses may be more susceptible to the impact of relatively low hCG concentrations during the late first trimester than female fetuses, considering the fact that mean hCG concentrations are lower in pregnancies with a male fetus [32, 33]. On the other hand, our findings show that the association of low hCG concentrations with decreased fetal growth is present in both female and male fetal sex. This suggests that low hCG concentrations during the first trimester have an impact on the fetal growth trajectory independent of fetal sex. Interestingly, the main difference between males and females in the current study was the association of high late first trimester hCG with growth acceleration during mid-pregnancy, which was only present in female fetuses. This might be due to the higher concentrations of hCG in female fetal sex, or due to fetal sex-specific differences in hCG isoforms [19]. Future studies are needed to investigate the mechanisms that underlie the differential effects of female fetal sex in the association of hCG and fetal growth in more depth. In addition, replication of fetal sex-specific differences in the association of hCG with fetal growth will be required, preferably in samples that have adequate power to test for higher order interactions.

The strengths of the current study include the availability of hCG concentrations as well as detailed fetal growth data with serial fetal weight measurements in a large population. The fact that hCG measurements were also available across a wide gestational time span, enabled us to observe a change in the association of hCG with fetal growth across gestational age. We were, however, limited by the fact that a single hCG measurement was available. As a consequence, we were not able to assess interindividual differences in the change of hCG concentrations during pregnancy. Also, the observational nature of this study does not allow for inference of causality and does not preclude the existence of residual confounding. As such, it is possible that it is the fetal size that affects maternal hCG concentration and/or that placental growth plays a confounding or mediating role. However, the latter seems less likely since the addition of placental weight to the model did not cause any meaningful changes in effect estimates.

In conclusion, we demonstrate that late first trimester hCG concentrations are associated with fetal growth and birth weight. In women with low hCG concentrations during the late first trimester, fetal growth is lower resulting in a lower birth weight. In female fetal-sex pregnancies, a high maternal hCG concentration at the end of the first trimester is associated with fetal growth acceleration. The underlying mechanism of these effects might involve a flawed protection from oxidative stress due to effects of hCG on arterial plug formation. Further research is necessary to investigate the causality of this association and the biological mechanism by which maternal hCG in the late first trimester affects fetal growth.

## Electronic supplementary material

Below is the link to the electronic supplementary material.

Supplementary material 1 (PPTX 260 kb)
