# Supplementary material for: Human chorionic gonadotropin (hCG) concentrations during the late first trimester are associated with fetal growth in a fetal sex-specific manner
Source: Eur J Epidemiol. 2016 Oct 5;32(2):135–44. doi: 10.1007/s10654-016-0201-3 (PMC5374189; doi:10.1007/s10654-016-0201-3)
Supplement: Supplementary file 1 — Supplementary material 1 (PPTX 260 kb) [file 10654_2016_201_MOESM1_ESM.pptx]

## Slide 1
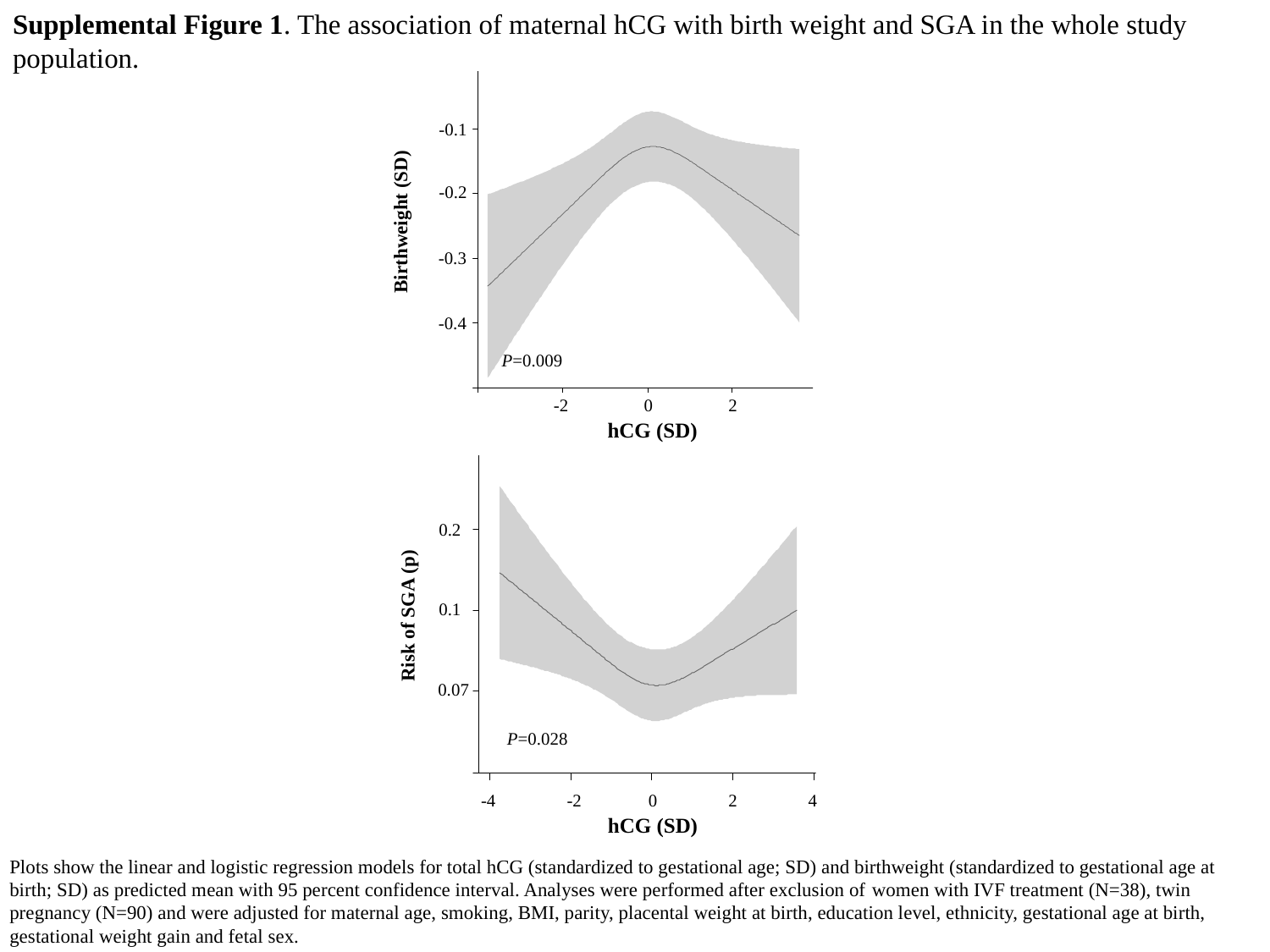

Supplemental Figure 1. The association of maternal hCG with birth weight and SGA in the whole study population.
Birthweight (SD)
-0.1
-0.2
-0.3
-0.4
P=0.009
 -2 0 2
hCG (SD)
Risk of SGA (p)
0.2
0.1
0.07
P=0.028
-4 -2 0 2 4
hCG (SD)
Plots show the linear and logistic regression models for total hCG (standardized to gestational age; SD) and birthweight (standardized to gestational age at birth; SD) as predicted mean with 95 percent confidence interval. Analyses were performed after exclusion of women with IVF treatment (N=38), twin pregnancy (N=90) and were adjusted for maternal age, smoking, BMI, parity, placental weight at birth, education level, ethnicity, gestational age at birth, gestational weight gain and fetal sex.

## Slide 2
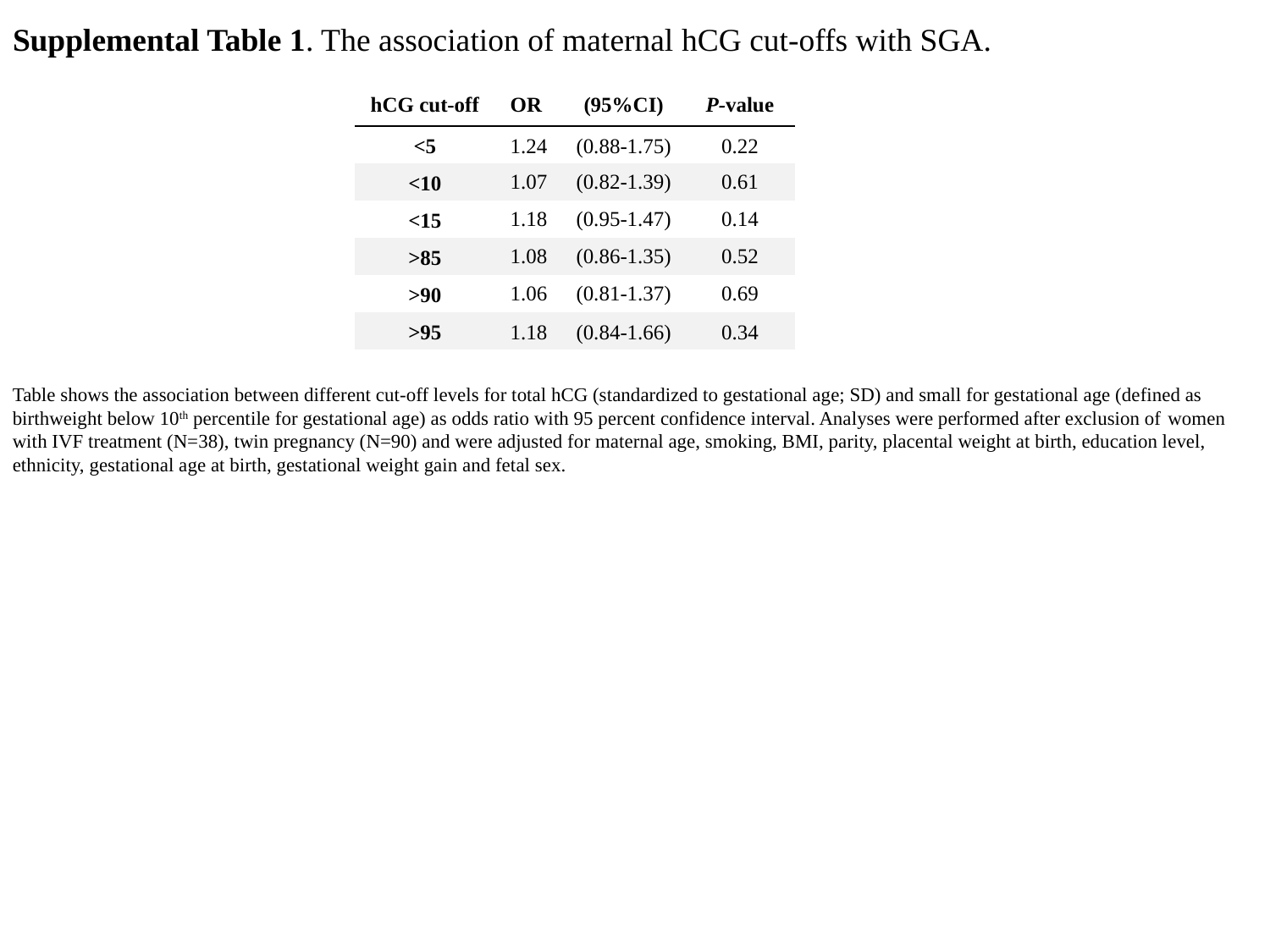

Supplemental Table 1. The association of maternal hCG cut-offs with SGA.
| hCG cut-off | OR | (95%CI) | P-value |
| --- | --- | --- | --- |
| <5 | 1.24 | (0.88-1.75) | 0.22 |
| <10 | 1.07 | (0.82-1.39) | 0.61 |
| <15 | 1.18 | (0.95-1.47) | 0.14 |
| >85 | 1.08 | (0.86-1.35) | 0.52 |
| >90 | 1.06 | (0.81-1.37) | 0.69 |
| >95 | 1.18 | (0.84-1.66) | 0.34 |
Table shows the association between different cut-off levels for total hCG (standardized to gestational age; SD) and small for gestational age (defined as birthweight below 10th percentile for gestational age) as odds ratio with 95 percent confidence interval. Analyses were performed after exclusion of women with IVF treatment (N=38), twin pregnancy (N=90) and were adjusted for maternal age, smoking, BMI, parity, placental weight at birth, education level, ethnicity, gestational age at birth, gestational weight gain and fetal sex.

## Slide 3
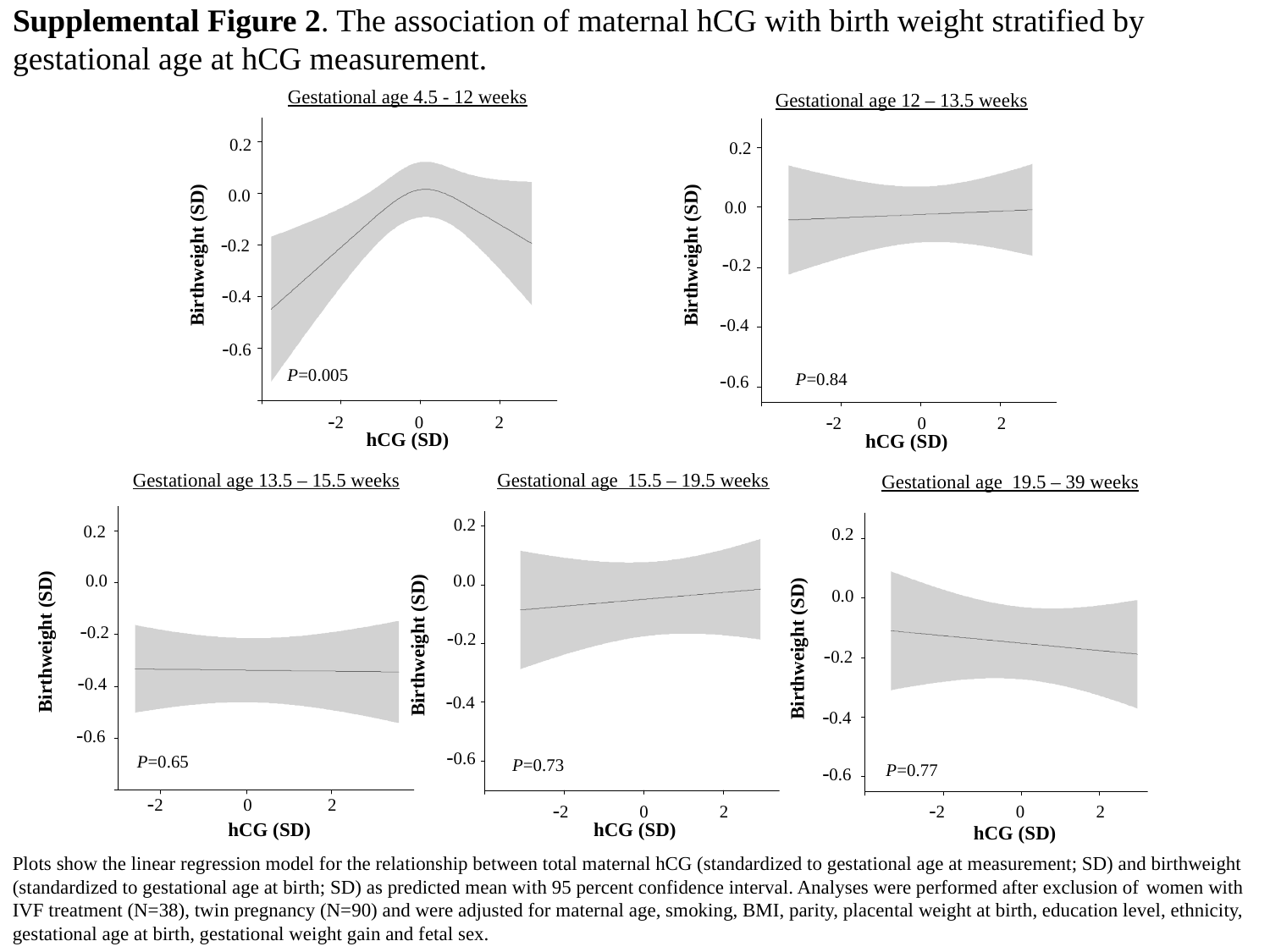

Supplemental Figure 2. The association of maternal hCG with birth weight stratified by gestational age at hCG measurement.
Gestational age 4.5 - 12 weeks
Gestational age 12 – 13.5 weeks
Birthweight (SD)
Birthweight (SD)
0.2
0.2
0.0
0.0
-0.2
-0.2
-0.4
-0.4
-0.6
P=0.005
-0.6
P=0.84
 -2 0 2
 -2 0 2
hCG (SD)
hCG (SD)
Gestational age 13.5 – 15.5 weeks
Gestational age 15.5 – 19.5 weeks
Gestational age 19.5 – 39 weeks
Birthweight (SD)
Birthweight (SD)
0.2
Birthweight (SD)
0.2
0.2
0.0
0.0
0.0
-0.2
-0.2
-0.2
-0.4
-0.4
-0.4
-0.6
-0.6
P=0.65
P=0.73
P=0.77
-0.6
 -2 0 2
 -2 0 2
 -2 0 2
hCG (SD)
hCG (SD)
hCG (SD)
Plots show the linear regression model for the relationship between total maternal hCG (standardized to gestational age at measurement; SD) and birthweight (standardized to gestational age at birth; SD) as predicted mean with 95 percent confidence interval. Analyses were performed after exclusion of women with IVF treatment (N=38), twin pregnancy (N=90) and were adjusted for maternal age, smoking, BMI, parity, placental weight at birth, education level, ethnicity, gestational age at birth, gestational weight gain and fetal sex.

## Slide 4
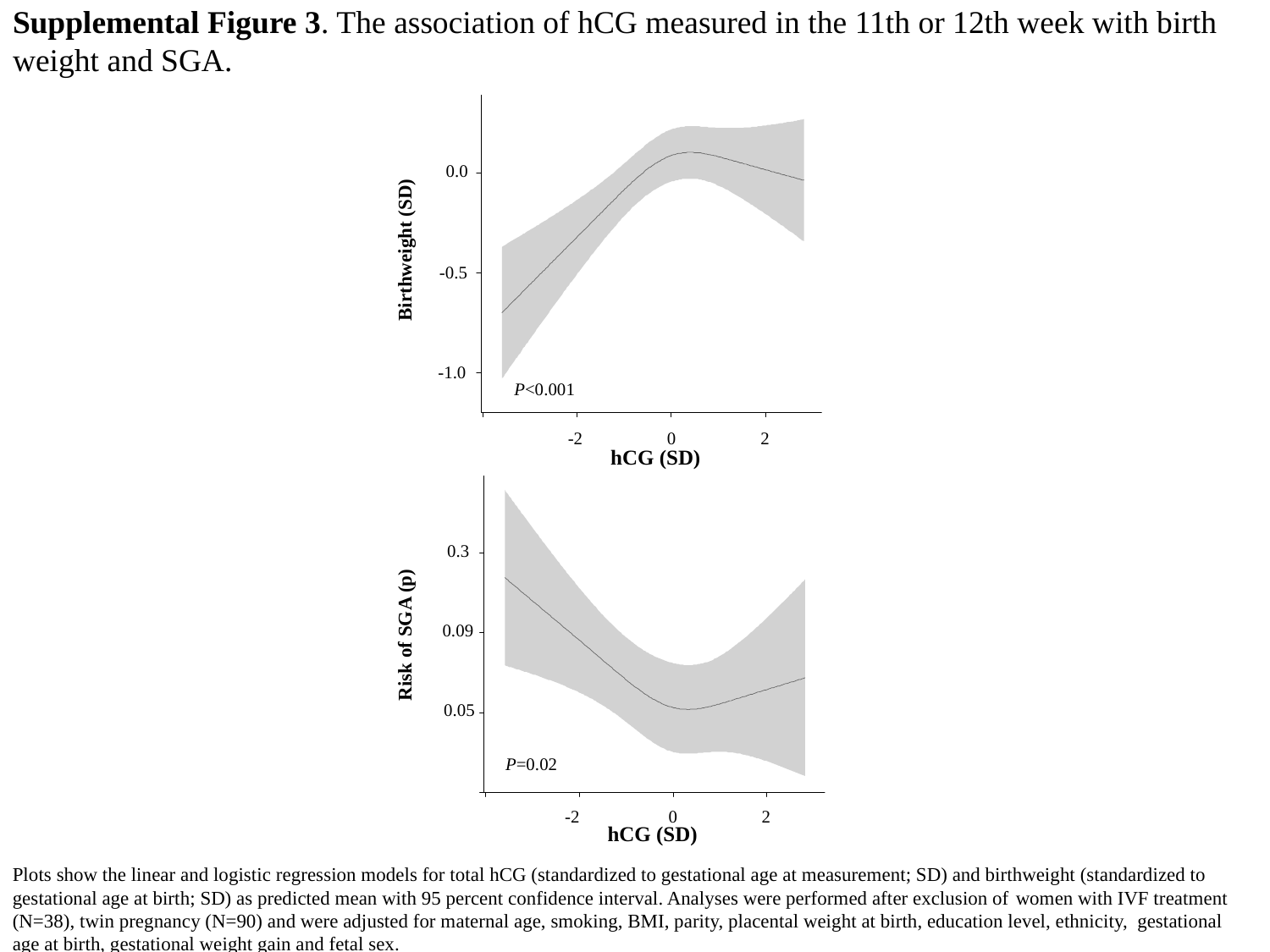

Supplemental Figure 3. The association of hCG measured in the 11th or 12th week with birth weight and SGA.
Birthweight (SD)
0.0
-0.5
-1.0
P<0.001
 -2 0 2
hCG (SD)
Risk of SGA (p)
0.3
0.09
0.05
P=0.02
 -2 0 2
hCG (SD)
Plots show the linear and logistic regression models for total hCG (standardized to gestational age at measurement; SD) and birthweight (standardized to gestational age at birth; SD) as predicted mean with 95 percent confidence interval. Analyses were performed after exclusion of women with IVF treatment (N=38), twin pregnancy (N=90) and were adjusted for maternal age, smoking, BMI, parity, placental weight at birth, education level, ethnicity, gestational age at birth, gestational weight gain and fetal sex.

## Slide 5
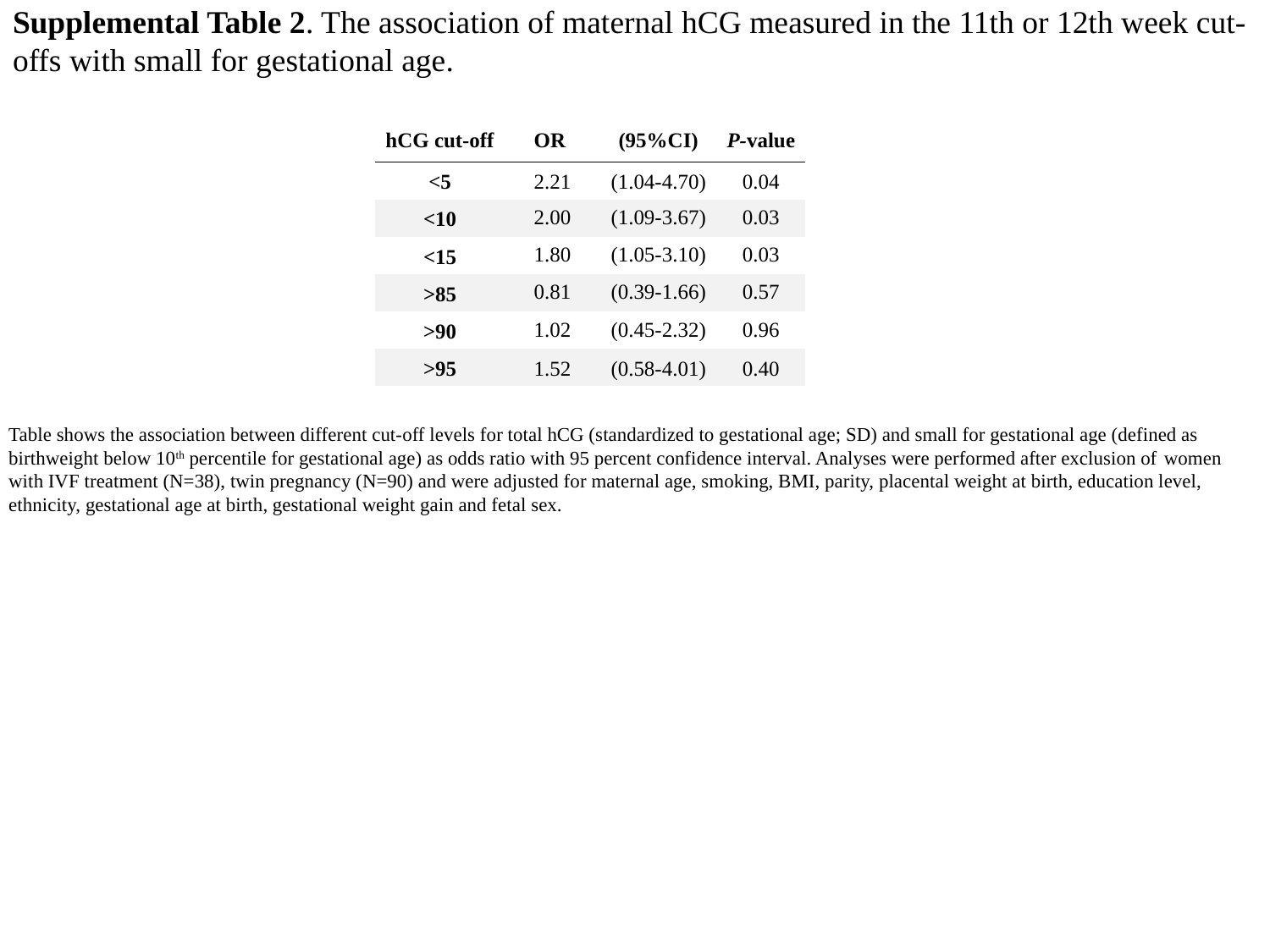

Supplemental Table 2. The association of maternal hCG measured in the 11th or 12th week cut-offs with small for gestational age.
| hCG cut-off | OR | (95%CI) | P-value |
| --- | --- | --- | --- |
| <5 | 2.21 | (1.04-4.70) | 0.04 |
| <10 | 2.00 | (1.09-3.67) | 0.03 |
| <15 | 1.80 | (1.05-3.10) | 0.03 |
| >85 | 0.81 | (0.39-1.66) | 0.57 |
| >90 | 1.02 | (0.45-2.32) | 0.96 |
| >95 | 1.52 | (0.58-4.01) | 0.40 |
Table shows the association between different cut-off levels for total hCG (standardized to gestational age; SD) and small for gestational age (defined as birthweight below 10th percentile for gestational age) as odds ratio with 95 percent confidence interval. Analyses were performed after exclusion of women with IVF treatment (N=38), twin pregnancy (N=90) and were adjusted for maternal age, smoking, BMI, parity, placental weight at birth, education level, ethnicity, gestational age at birth, gestational weight gain and fetal sex.

## Slide 6
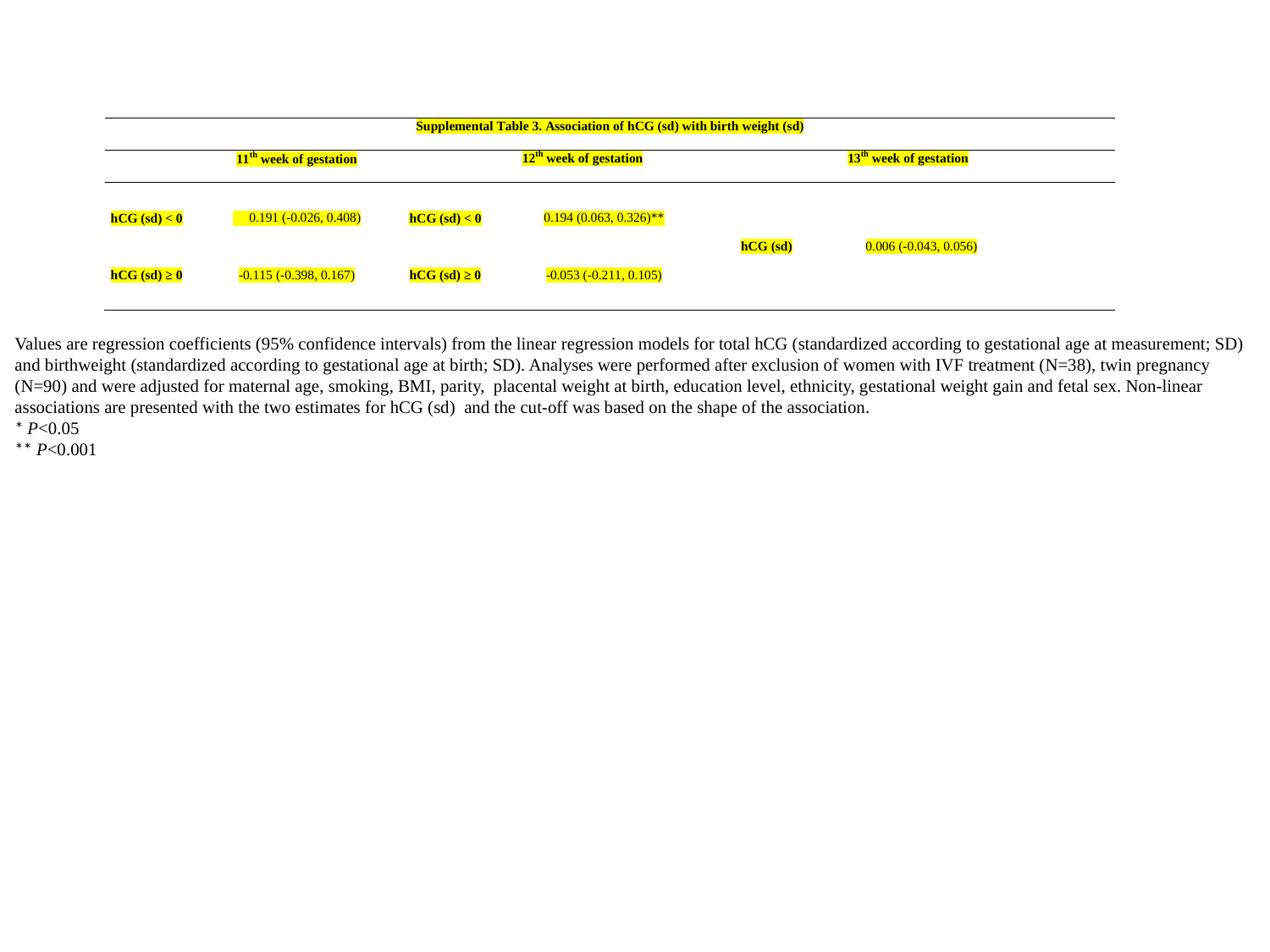

Values are regression coefficients (95% confidence intervals) from the linear regression models for total hCG (standardized according to gestational age at measurement; SD) and birthweight (standardized according to gestational age at birth; SD). Analyses were performed after exclusion of women with IVF treatment (N=38), twin pregnancy (N=90) and were adjusted for maternal age, smoking, BMI, parity, placental weight at birth, education level, ethnicity, gestational weight gain and fetal sex. Non-linear associations are presented with the two estimates for hCG (sd) and the cut-off was based on the shape of the association.
* P<0.05
** P<0.001

## Slide 7
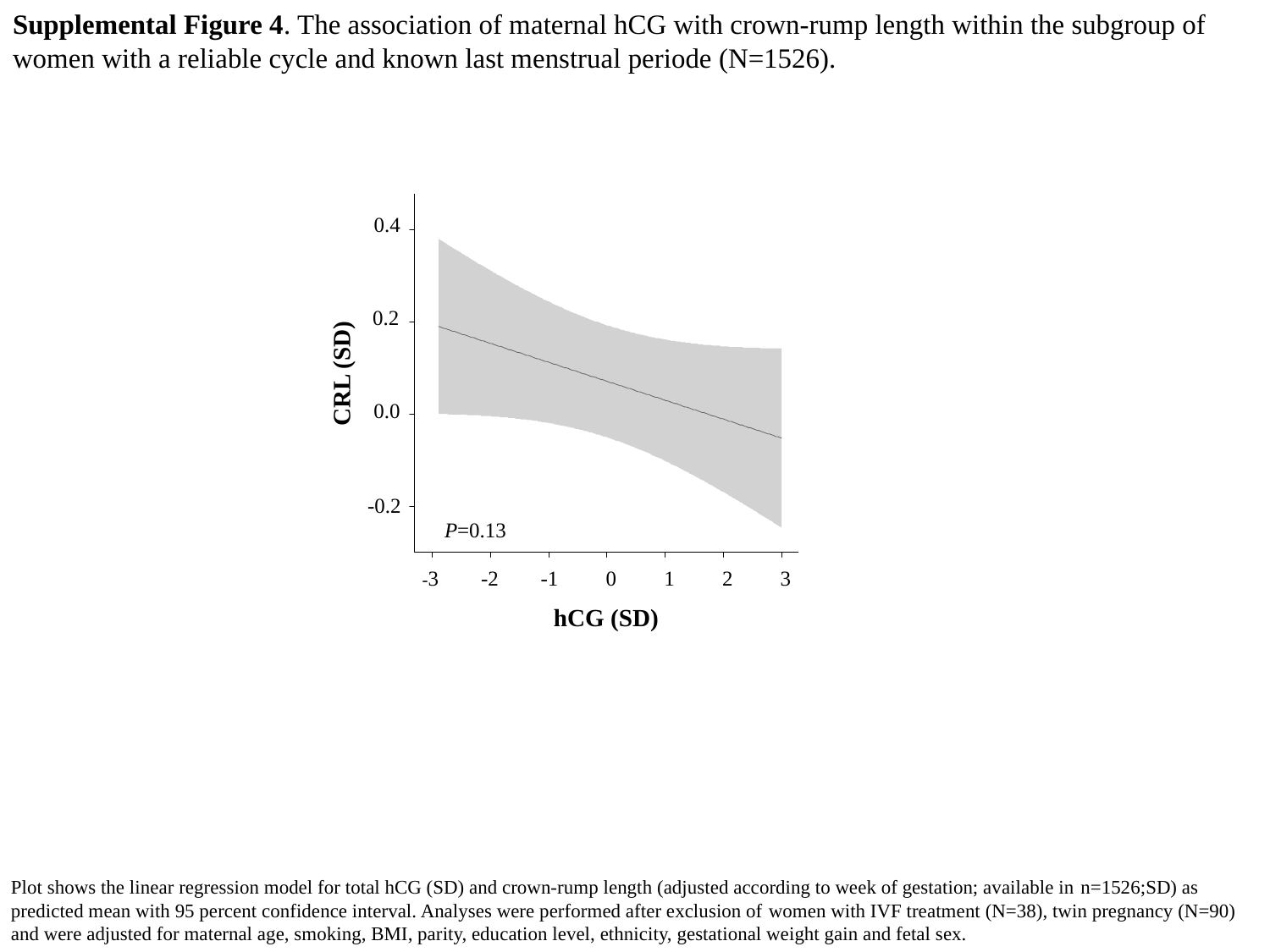

Supplemental Figure 4. The association of maternal hCG with crown-rump length within the subgroup of women with a reliable cycle and known last menstrual periode (N=1526).
CRL (SD)
0.4
0.2
 0.0
-0.2
P=0.13
 -3 -2 -1 0 1 2 3
 hCG (SD)
Plot shows the linear regression model for total hCG (SD) and crown-rump length (adjusted according to week of gestation; available in n=1526;SD) as predicted mean with 95 percent confidence interval. Analyses were performed after exclusion of women with IVF treatment (N=38), twin pregnancy (N=90) and were adjusted for maternal age, smoking, BMI, parity, education level, ethnicity, gestational weight gain and fetal sex.

## Slide 8
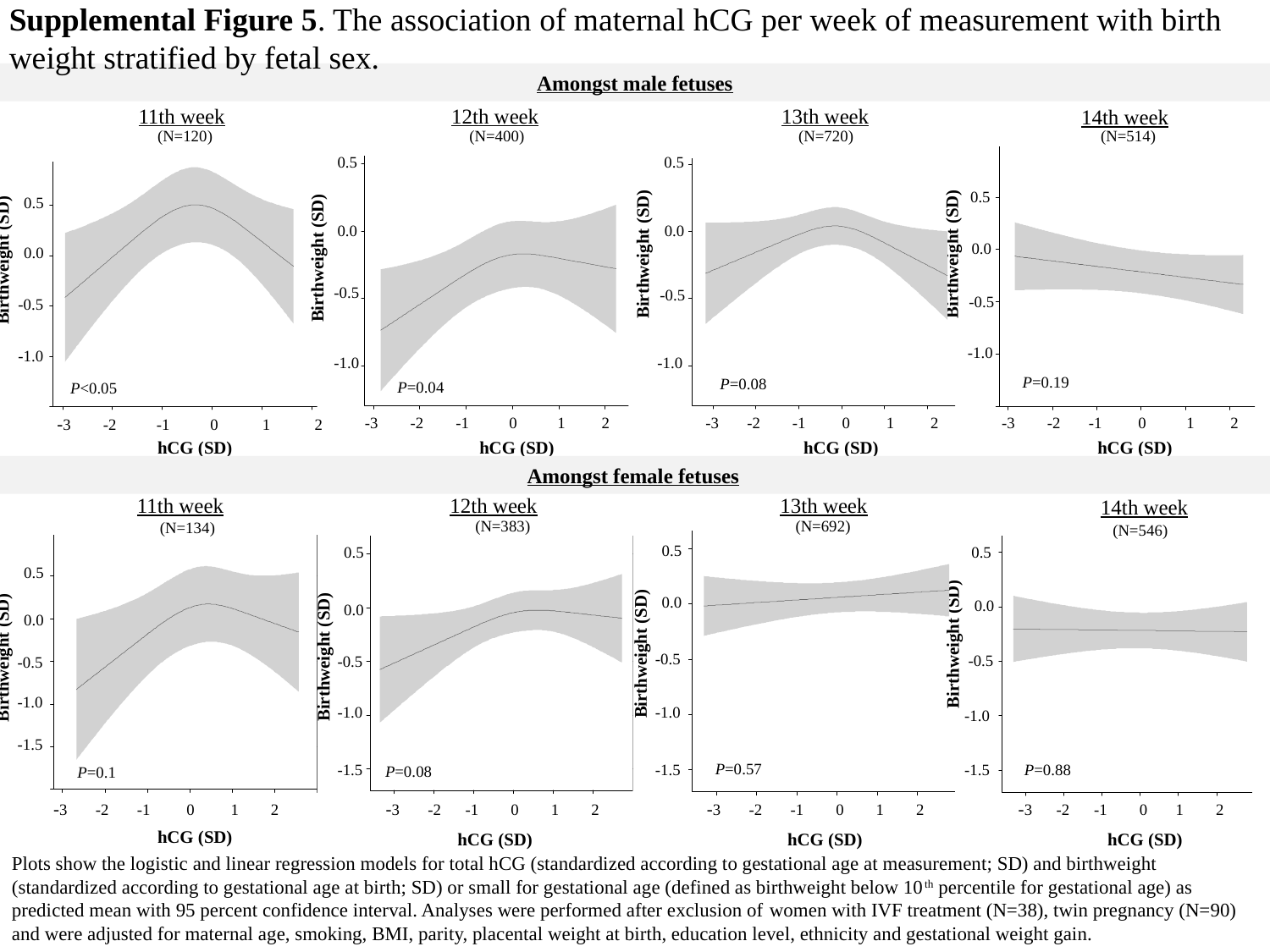

Supplemental Figure 5. The association of maternal hCG per week of measurement with birth weight stratified by fetal sex.
Amongst male fetuses
11th week
12th week
13th week
14th week
Birthweight (SD)
Birthweight (SD)
Birthweight (SD)
(N=120)
(N=400)
(N=720)
(N=514)
Birthweight (SD)
0.5
0.5
0.5
0.5
0.0
0.0
0.0
0.0
-0.5
-0.5
-0.5
-0.5
-1.0
-1.0
-1.0
-1.0
P=0.19
P=0.08
P=0.04
P<0.05
-3 -2 -1 0 1 2
 -3 -2 -1 0 1 2
 -3 -2 -1 0 1 2
 -3 -2 -1 0 1 2
hCG (SD)
hCG (SD)
hCG (SD)
hCG (SD)
Amongst female fetuses
11th week
12th week
13th week
14th week
Birthweight (SD)
(N=383)
(N=692)
(N=134)
(N=546)
Birthweight (SD)
Birthweight (SD)
Birthweight (SD)
0.5
0.5
0.5
0.5
0.0
0.0
0.0
0.0
-0.5
-0.5
-0.5
-0.5
-1.0
-1.0
-1.0
-1.0
-1.5
P=0.57
-1.5
-1.5
-1.5
P=0.88
P=0.08
P=0.1
 -3 -2 -1 0 1 2
 -3 -2 -1 0 1 2
-3 -2 -1 0 1 2
 -3 -2 -1 0 1 2
hCG (SD)
hCG (SD)
hCG (SD)
hCG (SD)
Plots show the logistic and linear regression models for total hCG (standardized according to gestational age at measurement; SD) and birthweight (standardized according to gestational age at birth; SD) or small for gestational age (defined as birthweight below 10th percentile for gestational age) as predicted mean with 95 percent confidence interval. Analyses were performed after exclusion of women with IVF treatment (N=38), twin pregnancy (N=90) and were adjusted for maternal age, smoking, BMI, parity, placental weight at birth, education level, ethnicity and gestational weight gain.

## Slide 9
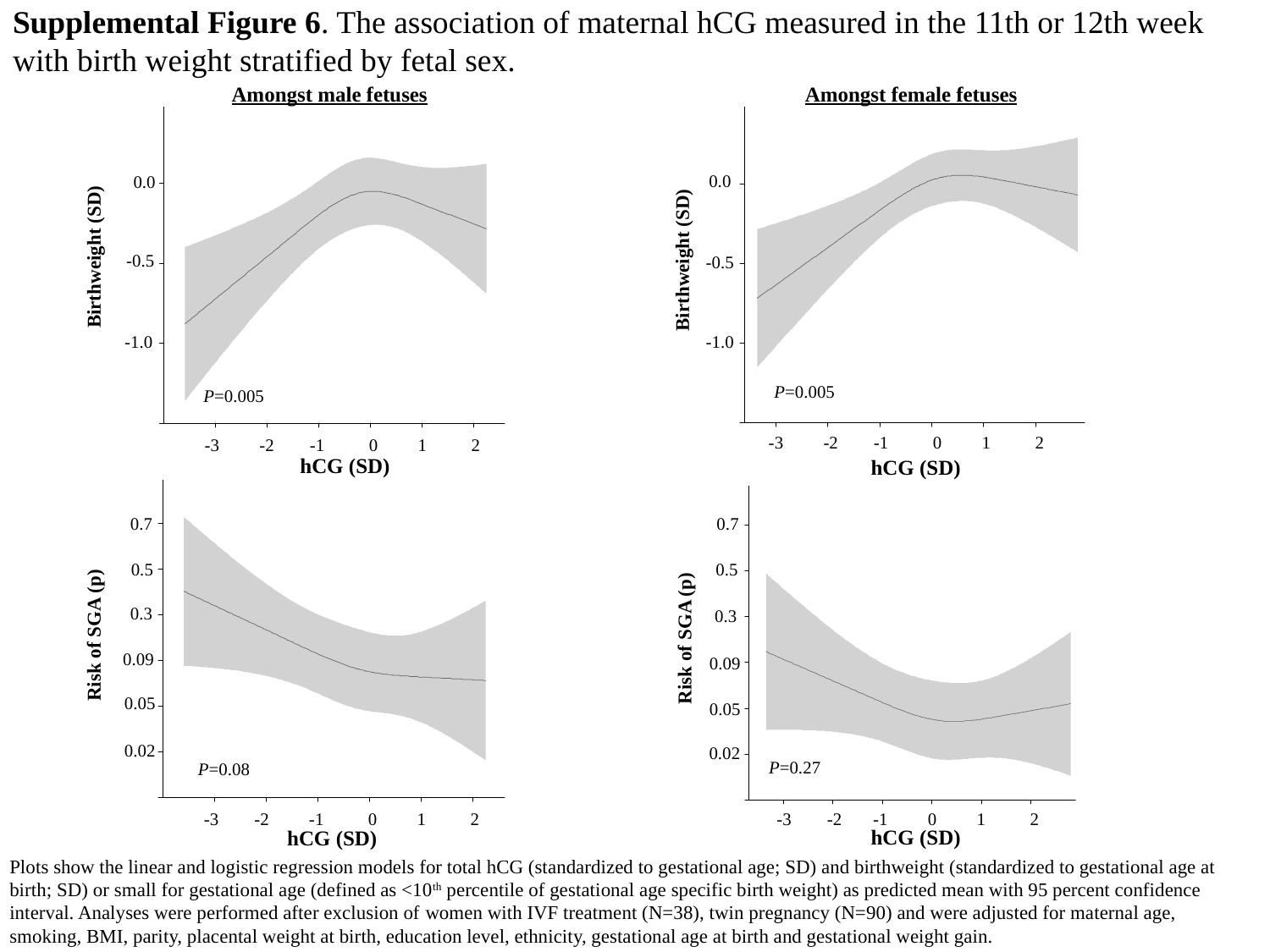

Supplemental Figure 6. The association of maternal hCG measured in the 11th or 12th week with birth weight stratified by fetal sex.
Amongst male fetuses
Amongst female fetuses
Birthweight (SD)
Birthweight (SD)
0.0
0.0
-0.5
-0.5
-1.0
-1.0
P=0.005
P=0.005
 -3 -2 -1 0 1 2
 -3 -2 -1 0 1 2
hCG (SD)
hCG (SD)
Risk of SGA (p)
Risk of SGA (p)
0.7
 0.7
 0.5
0.5
0.3
0.3
0.09
0.09
0.05
0.05
0.02
0.02
P=0.27
P=0.08
 -3 -2 -1 0 1 2
 -3 -2 -1 0 1 2
hCG (SD)
hCG (SD)
Plots show the linear and logistic regression models for total hCG (standardized to gestational age; SD) and birthweight (standardized to gestational age at birth; SD) or small for gestational age (defined as <10th percentile of gestational age specific birth weight) as predicted mean with 95 percent confidence interval. Analyses were performed after exclusion of women with IVF treatment (N=38), twin pregnancy (N=90) and were adjusted for maternal age, smoking, BMI, parity, placental weight at birth, education level, ethnicity, gestational age at birth and gestational weight gain.

## Slide 10
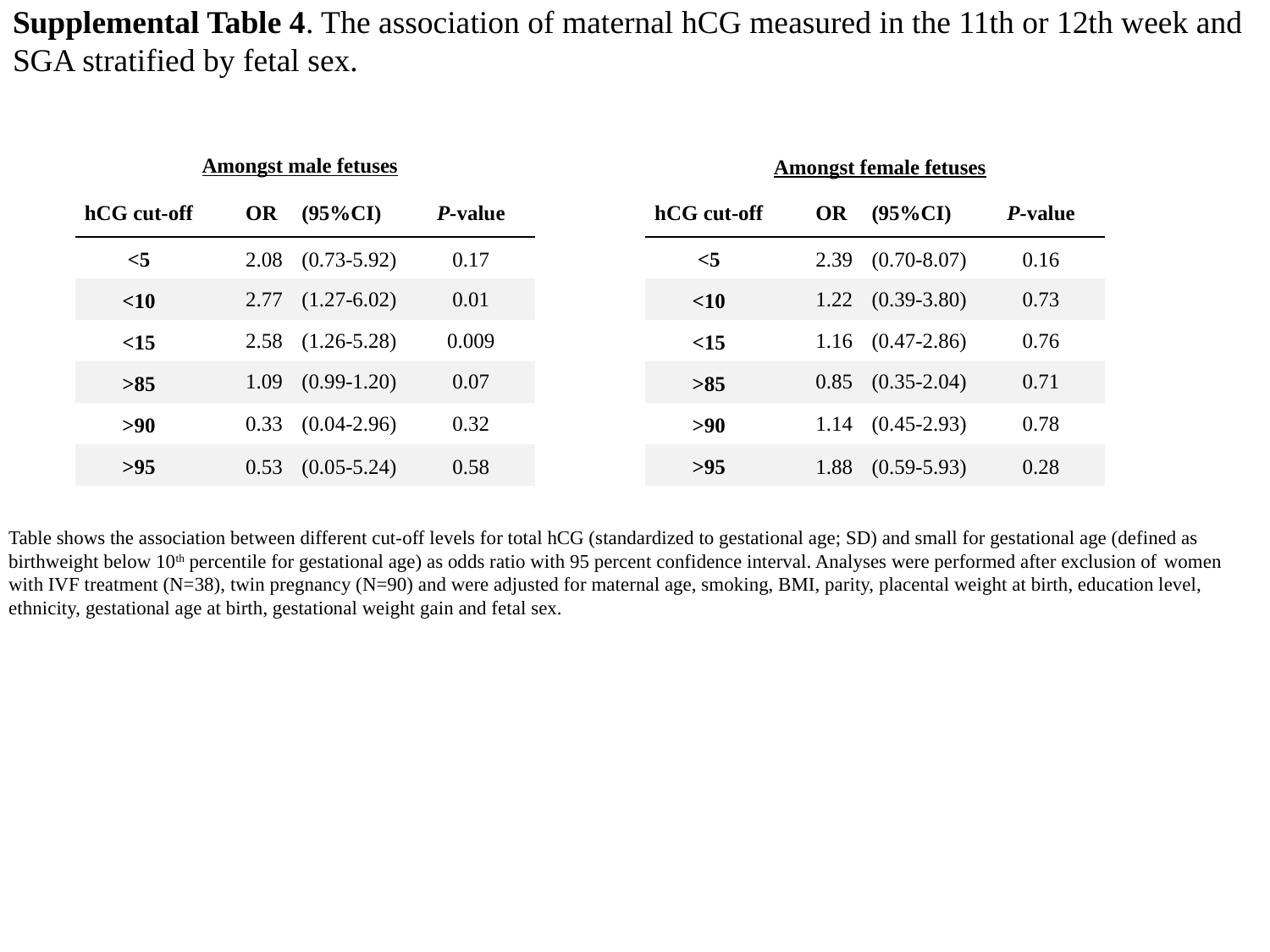

Supplemental Table 4. The association of maternal hCG measured in the 11th or 12th week and SGA stratified by fetal sex.
Amongst male fetuses
Amongst female fetuses
| hCG cut-off | OR | (95%CI) | P-value |
| --- | --- | --- | --- |
| <5 | 2.08 | (0.73-5.92) | 0.17 |
| <10 | 2.77 | (1.27-6.02) | 0.01 |
| <15 | 2.58 | (1.26-5.28) | 0.009 |
| >85 | 1.09 | (0.99-1.20) | 0.07 |
| >90 | 0.33 | (0.04-2.96) | 0.32 |
| >95 | 0.53 | (0.05-5.24) | 0.58 |
| hCG cut-off | OR | (95%CI) | P-value |
| --- | --- | --- | --- |
| <5 | 2.39 | (0.70-8.07) | 0.16 |
| <10 | 1.22 | (0.39-3.80) | 0.73 |
| <15 | 1.16 | (0.47-2.86) | 0.76 |
| >85 | 0.85 | (0.35-2.04) | 0.71 |
| >90 | 1.14 | (0.45-2.93) | 0.78 |
| >95 | 1.88 | (0.59-5.93) | 0.28 |
Table shows the association between different cut-off levels for total hCG (standardized to gestational age; SD) and small for gestational age (defined as birthweight below 10th percentile for gestational age) as odds ratio with 95 percent confidence interval. Analyses were performed after exclusion of women with IVF treatment (N=38), twin pregnancy (N=90) and were adjusted for maternal age, smoking, BMI, parity, placental weight at birth, education level, ethnicity, gestational age at birth, gestational weight gain and fetal sex.

## Slide 11
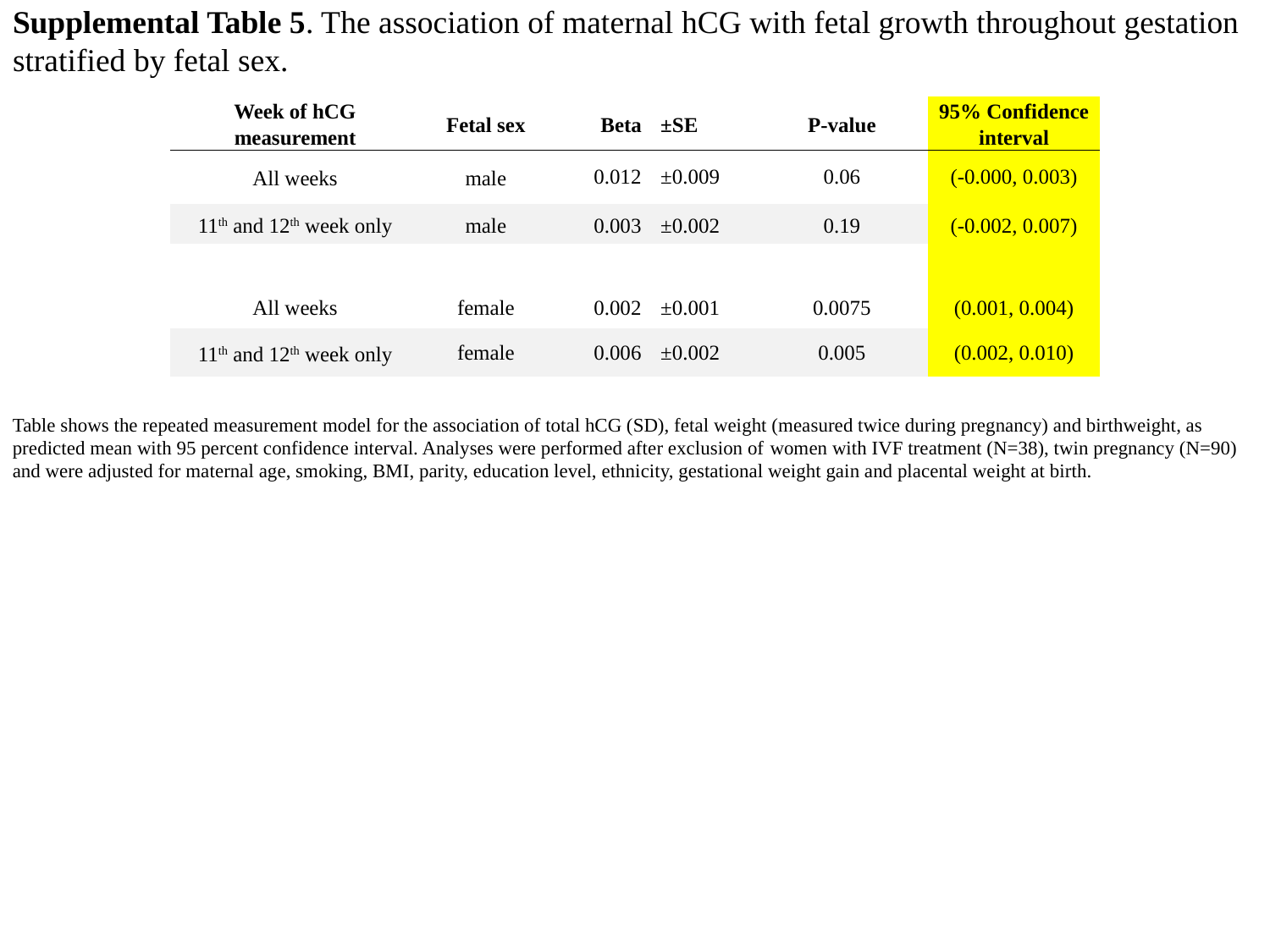

Supplemental Table 5. The association of maternal hCG with fetal growth throughout gestation stratified by fetal sex.
| Week of hCG measurement | Fetal sex | Beta | ±SE | P-value | 95% Confidence interval |
| --- | --- | --- | --- | --- | --- |
| All weeks | male | 0.012 | ±0.009 | 0.06 | (-0.000, 0.003) |
| 11th and 12th week only | male | 0.003 | ±0.002 | 0.19 | (-0.002, 0.007) |
| | | | | | |
| All weeks | female | 0.002 | ±0.001 | 0.0075 | (0.001, 0.004) |
| 11th and 12th week only | female | 0.006 | ±0.002 | 0.005 | (0.002, 0.010) |
Table shows the repeated measurement model for the association of total hCG (SD), fetal weight (measured twice during pregnancy) and birthweight, as predicted mean with 95 percent confidence interval. Analyses were performed after exclusion of women with IVF treatment (N=38), twin pregnancy (N=90) and were adjusted for maternal age, smoking, BMI, parity, education level, ethnicity, gestational weight gain and placental weight at birth.
